# Complicated Pyelonephritis Leading to Vertebral Osteomyelitis: Diagnostic and Therapeutic Considerations

**DOI:** 10.7759/cureus.84838

**Published:** 2025-05-26

**Authors:** Mustafa Ibraheem, Rafal Al-Rubaye, Ian Tanswell

**Affiliations:** 1 Internal Medicine, The Shrewsbury and Telford Hospital National Health Service (NHS) Trust, Telford, GBR; 2 Acute Medicine, The Shrewsbury and Telford Hospital National Health Service (NHS) Trust, Telford, GBR

**Keywords:** antibiotic therapy, case report, e. coli, pyelonephritis, vertebral osteomyelitis

## Abstract

This case illustrates the significant progression of pyelonephritis to vertebral osteomyelitis secondary to *Escherichia coli *bacteraemia, highlighting the potential for hematogenous dissemination from urinary tract infections to the spine. A female patient in her 60s initially presented with fever, nausea, and diarrhoea, subsequently diagnosed with *E. coli* bacteraemia from pyelonephritis. Despite initial clinical improvement with intravenous and oral antibiotics, her condition deteriorated over three hospital admissions, progressing to L1/L2 vertebral osteomyelitis complicated by bilateral psoas and epidural abscesses. Her functional status markedly declined from independent ambulation to requiring assistance for mobilization. This case emphasizes critical lessons regarding the complexity of determining optimal antibiotic therapy duration, especially when complicated by abscess formation and recurrent bacteraemia. It further underscores limitations associated with early transitions from intravenous to oral antibiotics in complicated vertebral infections, advocating heightened clinical vigilance and a multidisciplinary approach to prevent diagnostic delays and severe functional impairment.

## Introduction

Discitis often progresses to vertebral osteomyelitis, posing significant diagnostic and therapeutic challenges. Incidence rates range from 4 to 24 per million annually, rising due to improved diagnostics and ageing populations, particularly affecting those aged 60-75 years [1,2]. Pyogenic spondylodiscitis is predominant in Western countries, typically caused by *Staphylococcus aureus*, while *Mycobacterium tuberculosis* remains prevalent globally [3].

Clinical presentations, including fever and back pain, overlap with other conditions, often delaying diagnosis [4]. MRI with contrast is the diagnostic modality of choice due to its superior sensitivity and specificity [3,5]. Antibiotic therapy, guided by microbiological findings, is fundamental, though abscess drainage and surgical stabilization are often required for complicated cases [3].

## Case presentation

A 63-year-old female patient with type 2 diabetes mellitus with good glycemic control (HbA1C 44), hypothyroidism and a previous cerebrovascular accident presented initially with three days history of high-grade fever associated with nausea, and diarrhoea. Physical examination revealed significant suprapubic tenderness, while laboratory findings showed markedly elevated inflammatory markers (C-reactive protein (CRP), 205 mg/L, white blood count (WBC), 18×10⁹/L). Empirical intravenous (IV) antibiotics (piperacillin/tazobactam) were initiated for suspected urosepsis. During admission, the patient developed new-onset atrial fibrillation and required fluid resuscitation to maintain hemodynamic stability. Blood and urine cultures both yielded *Escherichia coli*. A computed tomography (CT) scan revealed localized pyelonephritis at the left kidney’s upper pole, prompting the addition of IV gentamicin based on culture sensitivities. Despite clinical improvement after seven days of IV antibiotics and subsequent discharge with oral amoxicillin, she developed urinary retention, necessitating catheterization. 

**Figure 1:**
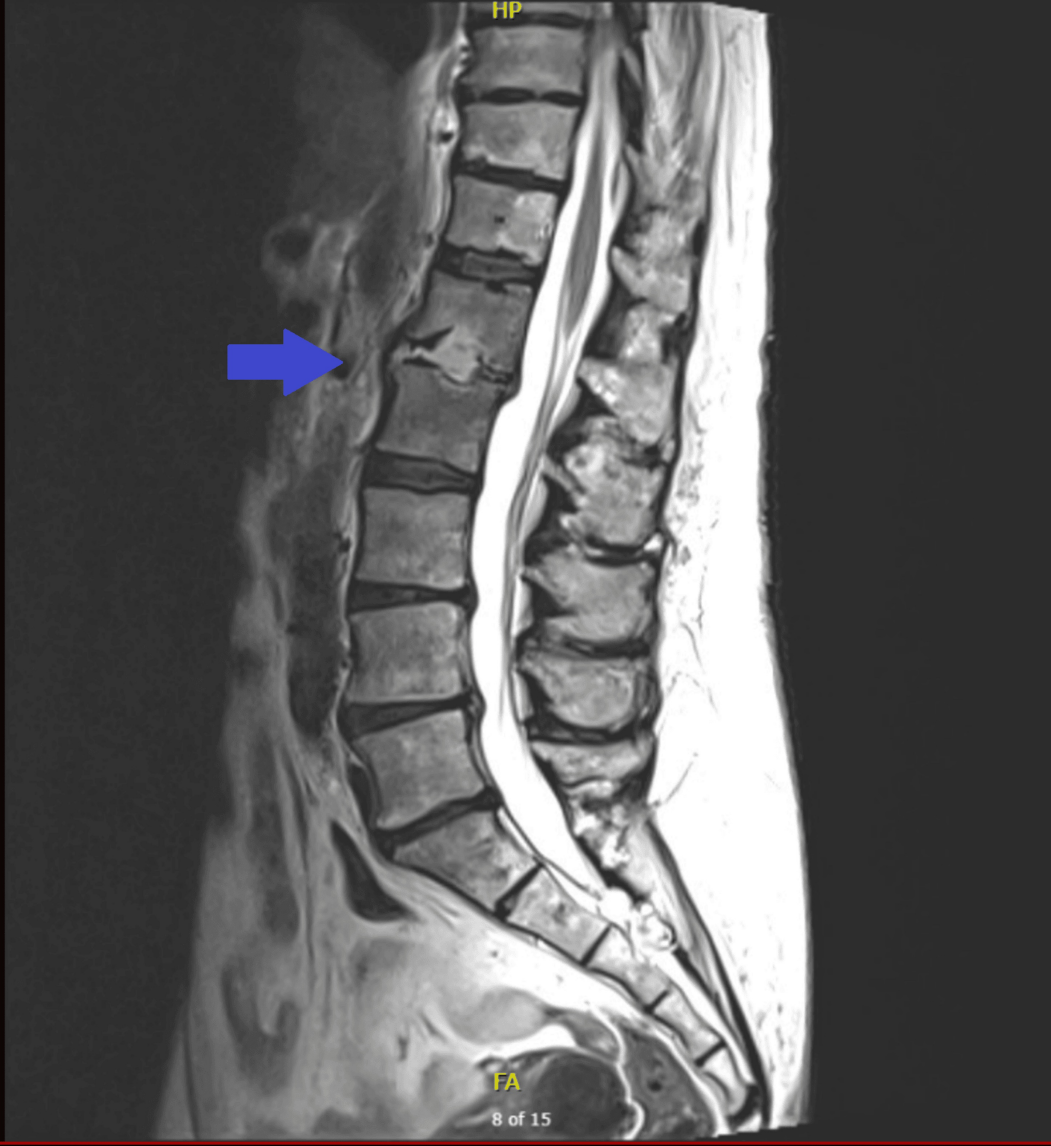
MRI lumber spine revealing abnormal high signal within the L1/L2 disc, erosions, and bone marrow oedema of the opposing vertebral endplates.

Eighteen days later, she returned with severe lower back pain localized to the lumbar spine, left flank discomfort, fatigue, and reduced appetite. She denied bowel incontinence and was still on urinary catheter. Examination demonstrated focal tenderness over the L2 vertebral level and bilateral weakness in hip flexion, with muscle power graded at 4/5. Power in the upper limbs was preserved, and sensation was intact in both upper and lower extremities. Laboratory tests indicated persistent inflammation (CRP 136 mg/L, WBC 15.9 × 10⁹/L), and blood cultures again yielded *E. coli* but no growth was found in the urine sample. MRI imaging (Figure 1) showed L1/L2 vertebral osteomyelitis complicated by small bilateral psoas and a small anterior epidural abscesses. 

She received a 10-day course of IV piperacillin/tazobactam, transitioning to four weeks of oral antibiotic as per local guidelines, with symptomatic improvement but significant functional decline requiring carer support upon discharge.

**Figure 2:**
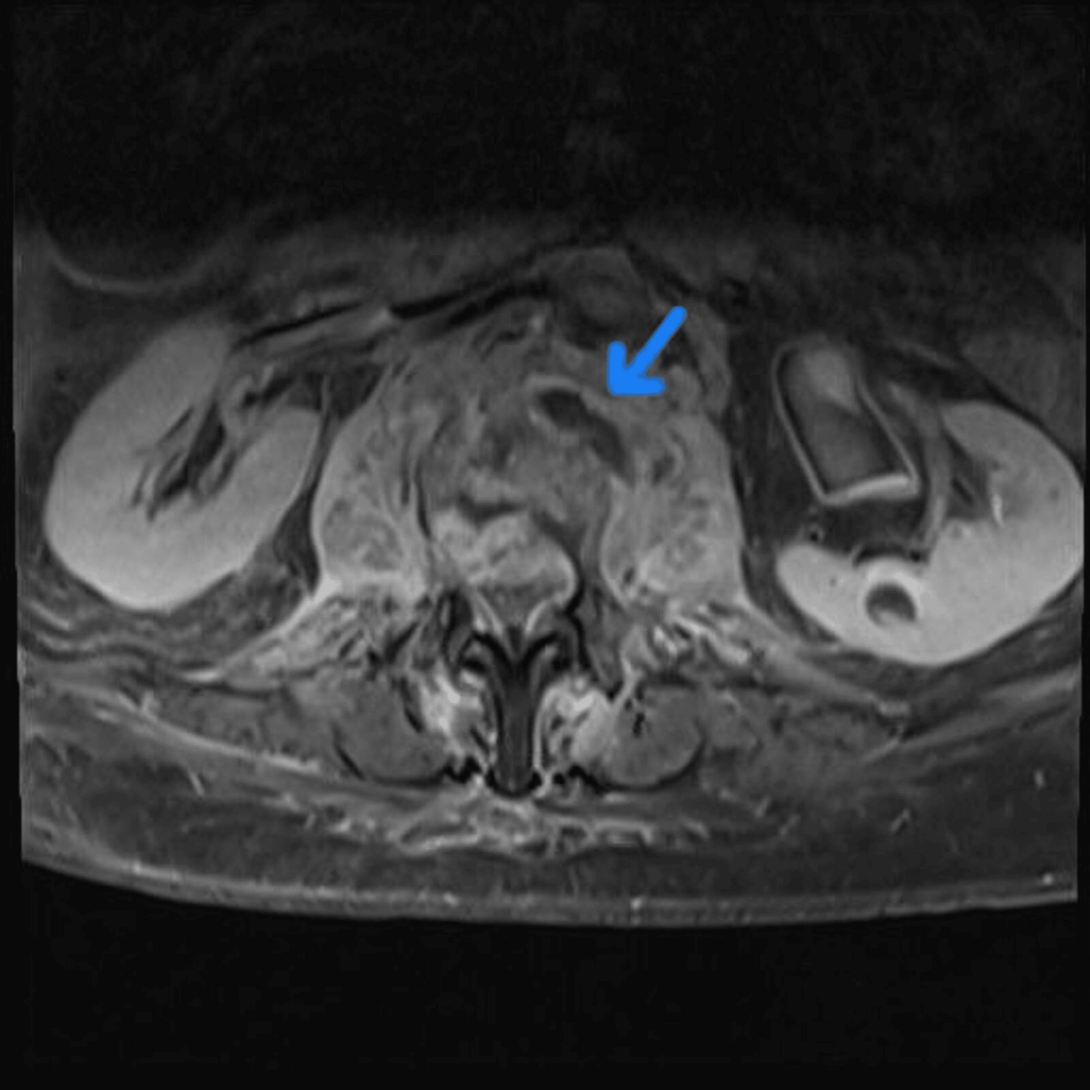
MRI lumber spine showing complete replacement of the L1 and L2 vertebral bodies by high signal, and a newly identified epidural abscess narrowing the spinal canal and indenting the conus medullaris.

Thirty-four days post-discharge, the patient experienced recurrent severe back pain, fever, and further deterioration of mobility. MRI (Figure 2) showed disease progression with enlargement of bilateral psoas and paravertebral abscesses. Blood and urine cultures remained negative. Given her frailty and significant functional impairment, conservative management was chosen after discussion with spinal surgery and microbiology, involving a prolonged six-week course of IV flucloxacillin, followed by another six weeks of oral flucloxacillin. Despite treatment adherence, the patient experienced profound functional decline, becoming nearly bedbound by discharge, underscoring the complexity and severity of managing recurrent vertebral osteomyelitis.

## Discussion

Urogenic spondylodiscitis is an uncommon yet recognized complication of urinary tract infections, representing approximately 6.2% of all pyogenic discitis cases [6]. The hematogenous dissemination of pathogens from the urinary tract to the vertebral column is well-established, although the exact mechanism remains speculative. Until recently, the Batson venous plexus was the most widely accepted theory explaining the hematogenous spread of infection from the pelvic organs to the spine. This valveless venous network connects the deep pelvic veins - such as those draining the bladder, prostate, and rectum - to the internal vertebral venous plexus, allowing for retrograde flow during episodes of increased intra-abdominal pressure [7,8]. This anatomical pathway plausibly explains the spread of *E. coli* from the patient's urinary tract to the lumbar vertebrae in this case. 

In this patient, positive blood and urine cultures consistently identified *E. coli* as the causative organism, although no biopsy was performed to confirm the diagnosis directly. Notably, *E. coli* is recognized as the leading cause of spontaneous gram-negative spondylodiscitis, accounting for 20.8% of all adult hematogenous cases [8]. Furthermore, gram-negative discitis disproportionately affects females and individuals with underlying risk factors, such as diabetes mellitus, both characteristics present in this patient [9].

Antimicrobial therapy remains the cornerstone of vertebral osteomyelitis management. Current guidelines advocate an initial course of intravenous (IV) antibiotics for at least two weeks, transitioning to oral therapy when clinically appropriate [10]. However, the optimal duration of IV antibiotic therapy in complex cases involving abscess formation remains uncertain. The Oral versus Intravenous Antibiotics for Bone and Joint Infection (OVIVA) trial demonstrated the non-inferiority of oral antibiotics compared to IV therapy for uncomplicated bone and joint infections, including vertebral osteomyelitis; however, its findings are less applicable to cases with significant abscess formation. Additionally, an observational study by Roblot et al. found no difference in relapse or mortality between shorter (≤six weeks) and prolonged (≥six weeks) antibiotic treatments [11]. Consequently, this case suggests that prolonged IV antibiotic therapy might have been more appropriate given the patient's clinical complexity and recurrent presentations.

The decision between surgical and conservative management for pyogenic spondylodiscitis remains debated. While conservative antibiotic management typically serves as the initial approach, early surgical intervention has shown improved outcomes concerning mortality, relapse rates, and hospital length of stay, as evidenced by a recent meta-analysis [12,13]. In this patient's case, however, conservative management was favoured due to her significant functional decline, limited physiological reserve, and multiple comorbidities. Similarly, percutaneous drainage of the bilateral psoas abscesses was deferred to avoid further compromising her frail condition.

## Conclusions

In conclusion, this case highlights important considerations in determining optimal antibiotic strategies for managing complicated vertebral osteomyelitis, emphasizing the potential necessity of extended intravenous therapy and tailored individual treatment plans. It underscores the critical need for carefully evaluating clinical guidelines within the complexities of each patient's presentation, balancing aggressive therapeutic interventions with patient frailty to minimize morbidity and functional decline.
